# Horizontal vascular-stalked split osteotomy (HVSO): a bone augmentation technique for the atrophic jaw– a retrospective cohort study in 29 patients

**DOI:** 10.1186/s40729-025-00608-8

**Published:** 2025-03-07

**Authors:** Dirk Nolte, Johannes Angermair, Heidi Bradatsch, Rolf Ewers, Michael Alfertshofer, Robert Linsenmann, Sven Otto, Karin Christine Huth

**Affiliations:** 1 Private Practice for Oral and Maxillofacial Surgery, Munich, Germany; 2 Department of Oral and Maxillofacial Surgery, University Medical Center Munich, Munich, Germany; 3 Private Practice for Oral Surgery, Wiesbaden, Germany; 4 University Hospital for Cranio- Maxillofacial and Oral Surgery, Medical University Vienna, Vienna, Austria; 5 Department of Conservative Dentistry and Periodontology, University Hospital Munich, LMU Munich, Munich, Germany; 6 Department of Oral and Maxillofacial Surgery, Charité– Universitätsmedizin Berlin, Corporate member of Freie Universität Berlin, Humboldt-Universität zu Berlin, Berlin Institute of Health, Berlin, Germany

**Keywords:** Bone augmentation, Split osteotomy, Implantology, Dental implants, Oral surgery

## Abstract

**Background:**

Implant therapy in the advanced atrophic jaw remains challenging in oral and maxillofacial surgery. Hence, a plethora of different augmentation procedures to increase bone volume in the maxilla and mandible have been published. Horizontal vascular-stalked split osteotomy (HVSO) represents a safe and effective approach for the three-dimensional jaw augmentation since it combines maximum vascularization through lingual or palatinal periosteal stalking with reduced grafting morbidity.

**Objective:**

To analyze the efficacy of HVSO for implantation therapy in atrophic jaws by assessing vertical bone gain and implant survival rates.

**Materials and methods:**

A total of *n* = 29 patients (14 females, 15 males) with a mean age of 55.4 ± 10.0 years and reduced volume of the alveolar ridge were retrospectively analyzed after treatment with 34 HVSOs in the maxilla and mandible. After controlled clinical follow-up of six months after augmentation, enossal implantation of 79 implants (maxilla 45, mandible 34) was performed. A standardized two-dimensional radiological assessment with panoramic tomography (OPTG) of the augmented bone height and clinical evaluation of the implants was performed over a mean follow-up period of 2.3 years.

**Results:**

HVSO resulted in a significant increase in vertical bone height by 4.4 mm ± 2.0 mm (mean vertical gain: +59.4%) with + 101% in the maxilla and + 27.5% in the mandible directly after the procedure (T1), with both *p* < 0.001. After a mean observation period of 2.3 years bone height remained stable with a total gain of 41.4% (maxilla: 72.6%, mandible: 18.6%), with *p* < 0.001 and *p* = 0.001, respectively. Overall implant survival rate was 91% (maxilla: 89%; mandible: 94%).

**Conclusion:**

HVSO reliably supports significantly enhanced vertical bone height with long-term stable results, thereby facilitating successful implantation in atrophic jaws with high implant survival rates observed over an extended follow-up period.

**Clinical Trial Number:**

Not applicable as the study was no clinical trial.

## Introduction

Recent statistics highlight a growing trend for dental implants as an effective solution for the replacement of missing teeth, with millions of individuals worldwide undergoing this treatment each year [1–3]. This surge underscores the increasing public perception of the benefits and potential of dental implants in enhancing functionality, oral health, and overall quality of life [4, 5]. 

The clinical outcomes of dental implant therapies are intricately linked to the condition of the jawbone. A plethora of studies has reported on the correlation between bone structure and success of dental implant therapy: Strong bone architecture and thickness have been reported to be beneficial while bone volume loss and reduction of stability, as seen in jaw atrophy, is detrimental to the success and survival of dental implant therapy [6–9]. Concomitant with the increasingly aging population, the prevalence of jaw atrophy, characterized by the loss of bone volume in the upper (maxilla) and/or lower (mandible) jaw, rises likewise [10–12]. This condition affects a significant number of individuals seeking dental implant therapy and can complicate the implantation process substantially, requiring more complex surgical interventions to build up bone and create a solid base prior to the placement of dental implants [13, 14]. However, surgical bone augmentation techniques are often characterized by their traumatic nature with inherently associated surgical complications and postoperative morbidity [15–17]. 

The authors herein propose the Horizontal Vascular-Stalked Split Osteotomy (HVSO) as an innovative technique tailored to address the clinical challenges of dental implant placement in atrophic jaws. Similar to previously reported interpositional bone augmentation techniques [18, 19], HVSO addresses the demand for minimally invasive approaches by prioritizing the preservation of vascular integrity in the mobilized bone segments. This method aims to reduce the trauma and postoperative morbidity often associated with traditional grafting procedures, offering a safer and more effective solution for jawbone augmentation. Latter approaches have been reported to be associated with considerable patient discomfort due to their higher degrees of invasiveness [17, 20, 21]. Previously, a shift towards vascular preservation in jawbone augmentation has been noted in the literature. To expand on this concept even further, the HVSO introduces a technique aiming to maximize vascular blood supply through lingual or palatinal periosteal stalking, thereby minimizing the need for external bone graft materials. This innovative approach not only aims to enhance bone augmentation outcomes, but also aligns with the evolving principles of minimally invasive surgery, offering new hope to those previously considered unsuitable candidates for implant therapy with extensive jaw atrophy.

In this study, retrospective analysis of the gain in vertical bone height and the survival rates of dental implant therapy was performed after jawbone augmentation employing HVSO to assess its role in the treatment of patients with severe jaw atrophy.

## Materials and methods

### Study design

This study was designed as a retrospective clinical study to assess the clinical and radiological outcome of the horizontal vascular-stalked split osteotomy (HVSO) and of subsequent implant therapy. The patients included in this retrospective analysis were treated employing this technique in a private clinic for oral and maxillofacial surgery in Munich, Germany in the period from February 2007 to July 2015. Prior to inclusion into the study, patients were informed about the use of their data for academic purposes and written informed consent was obtained. The study was approved by the Institutional Review Board of Ludwig-Maximilians-University Munich (IRB protocol number 24–0912). All patients were treated in accordance with regional laws, good clinical practice and in adherence to the Declaration of Helsinki (1996) [22]. 

### Patient sample

Adult patients (completed facial growth) were included upon individual examination resulting in the indication for the HVSO procedure. This was considered if there was an atrophied, partially edentulous alveolar ridge, in which bone height was insufficient (i.e., < 3 mm) for a one-stage implantation or in which a sinus lift procedure was not possible or useful for reasons of severe crestal vertical bone deficit. Severe jaw atrophy was defined clinically as the absence of sufficient bone volume to allow for primary implant placement, corresponding to Class IV bone in the mandible and Class V bone in the maxilla according to the Cawood and Howell classification [23]. Herein, the maxilla typically presented with very low bone heights in the range of 2–5 mm, while the mandible, despite higher mean bone heights, was often narrow and pointed, making implant placement impossible without horizontal augmentation, even with diameter-reduced implants [24]. This anatomical duality highlights the necessity of HVSO to enable stable implantation in cases that are no longer amenable to conventional implantation.

Exclusion criteria involved general contraindications for dental and surgical treatment, such as autoimmune diseases, prior bisphosphonate or corticosteroid therapy, as well as a history of malignancies in the head and neck region requiring chemotherapy or radiotherapy. The study size of 29 patients was determined by the availability of cases meeting the strict inclusion criteria within the defined study period, reflecting the rarity and complexity of the condition being treated.

### Surgical bone augmentation procedure

**Fig. 1:**
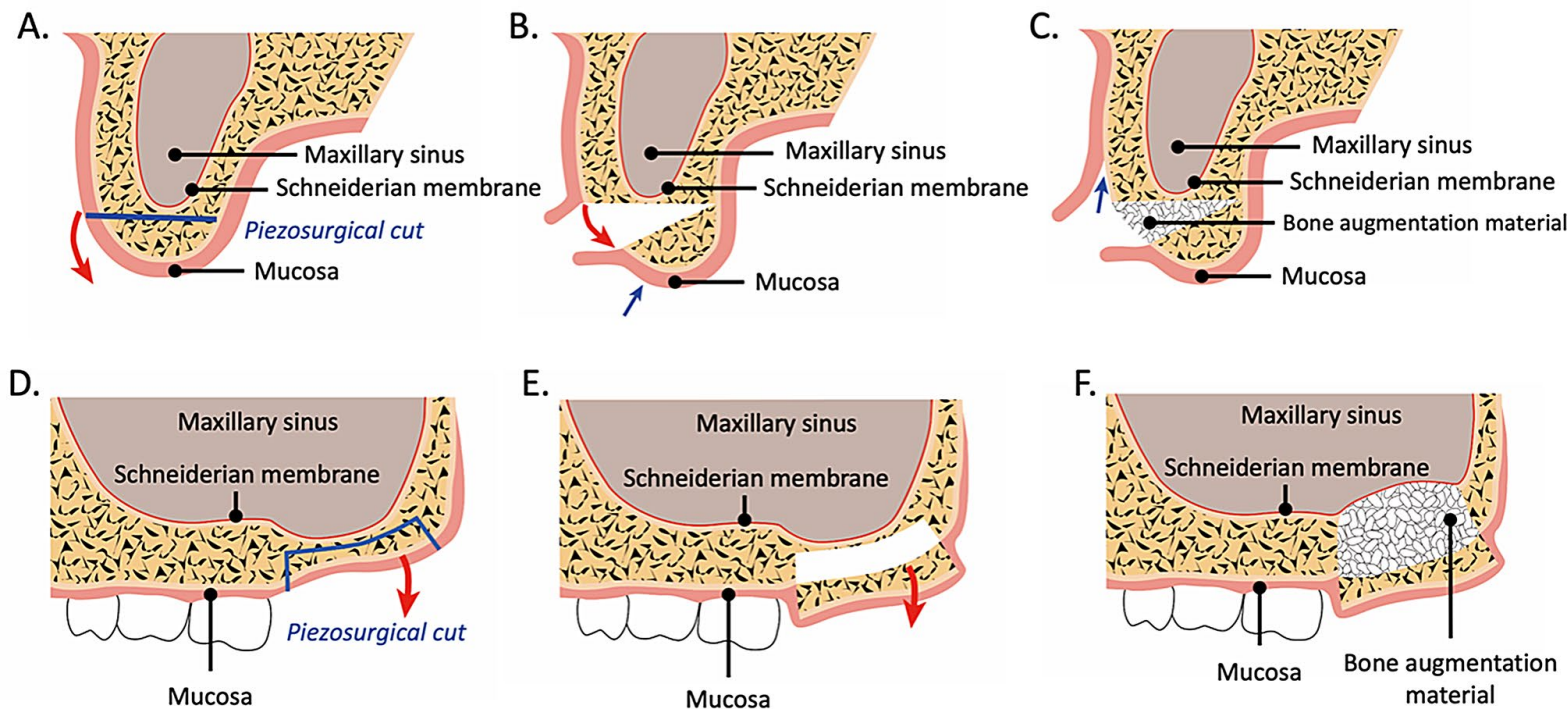
Schematic illustration of the surgical technique of the HSVO in the upper molar region (coronal [**A**.-**C**.] and sagittal [**D**.-**F**.] sections). **A**.: Paramarginal incision (red line) followed by horizontal split osteotomy (blue line) using piezosurgery at the height of the mucogingival border; **B**.: Bone splitting using chisels and mobilization in crestal and palatinal direction (red line) to augment both horizontal and vertical bone volume; **C**.: Insertion of grafting material, mucosal mobilization using periostal incision (blue line) to achieve tension-free wound closure. **D**.: Horizontal osteotomy with vertical osteotomy lines mesially and distally in tunneling technique (blue line); **E**.: Mobilization of the tissue pedicled bone fragment (red line) **F**.: Interpositional graft in situ, mobilization using periostal incision and tension-free wound closure

HVSO describes a bone splitting technique particularly suitable for combined horizontal and vertical alveolar bone deficiencies. The mucosal incision is made below the mucogingival border line in the non-attached gingiva. After careful rising a full-thickness mucoperiosteal flap covering the bone segment to be elevated as much as possible, osteotomies were performed with a piezosurgical instrument (Piezosurgery, Mectron S.p.A., Carasco, Italy) in horizontal direction along the entire length of the edentulous area to be augmented and two vertical directions mesially and distally in form of the so-called garage door incision. The use of a piezosurgical instrument enables a minimally invasive tunneling preparation, in which the vascular trunks are spared. Due to the risk of heating, particularly careful preparation under constant cooling with sterile physiological saline is mandatory [25]. The horizontal osteotomy line is bordered by the two vertical tunneling osteotomies mesially and distally (Fig. 1).

**Fig. 2:**
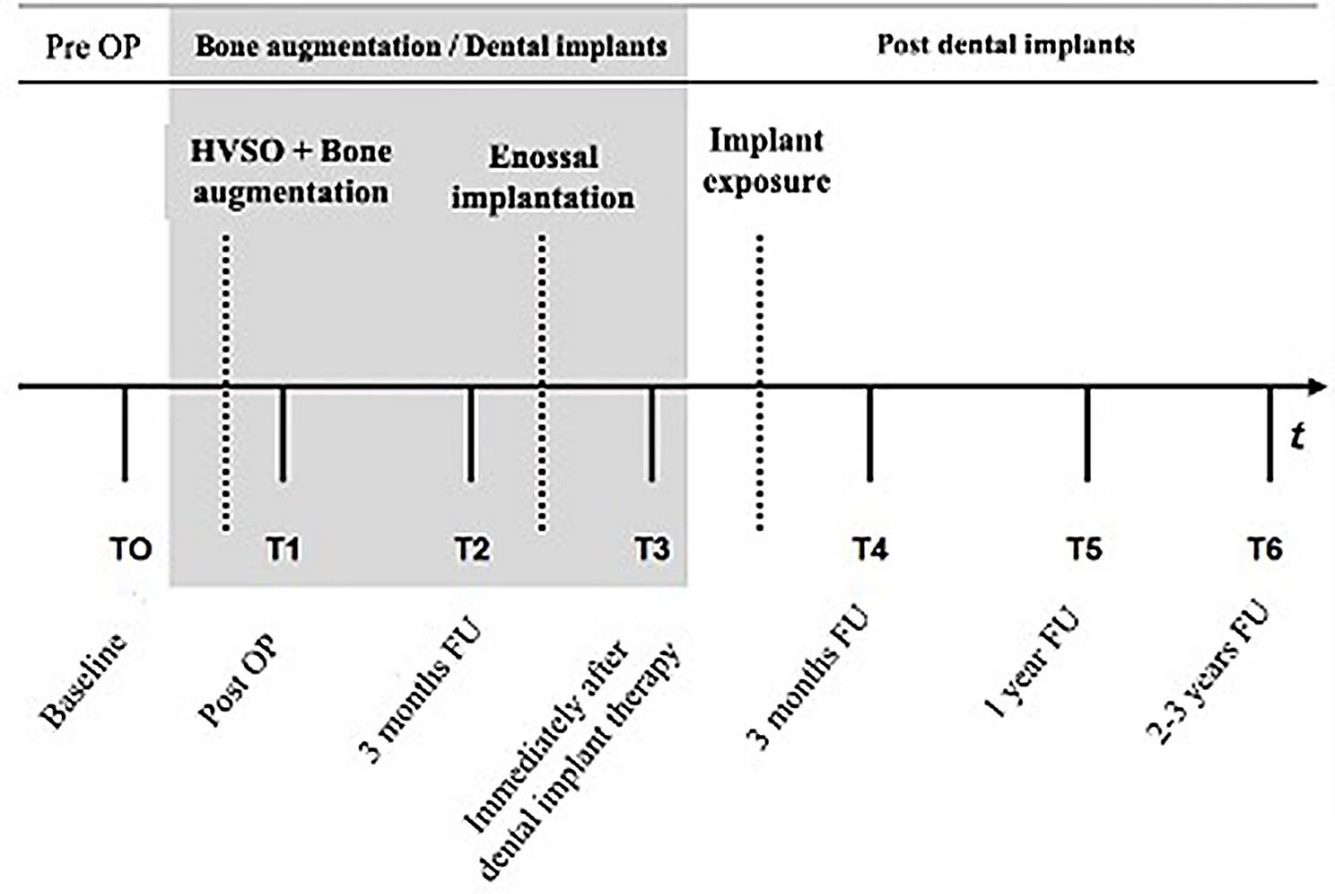
Study design and clinical follow-up (FU) protocol. Clinical and radiographic examination parameters were collected at fixed time points (T0, T1,…T6)

The gingiva is minimally detached from the bone and the bone split is finalized witha chisel blow to facilitate the lifting of the stalked bone fragment. The bony gap is filled with augmentation material of either allogenic, alloplastic or combined allogenic and autologous origin. The technique can, if necessary, be combined with sinus lifting procedures in the maxilla, particularly during implantation, to enhance apical bone density if required. Due to the fragility and risk of compromising the mobilized bone fragment, osteosynthesis plates were avoided, with clinically sufficient stabilization achieved using graft material alone instead. The grafted region is covered buccally using a resorbable collagen membrane (Bio-Gide^®^, Geistlich Pharma AG, Wolhusen, Switzerland) to stabilize the blood coagulum and grafting material in the prepared cavity and to prevent resorption. To achieve tension free wound closure periosteal-releasing incisions are performed and the mucoperiosteal flap is fixed using resorbable sutures (Vicryl 4 − 0 Ethicon, Johnson & Johnson, Raritan, NJ, USA). Patients were scheduled for suture removal after one week. Re-entry was performed 3 months after the augmentation procedure and the further clinical and radiological follow-up was planned according to a standardized study protocol (Fig. 2).

Four implant systems were used: 75% (59) implants from Straumann (Straumann^®^ Dental Implant System, Basel, Switzerland), 16% (13) implants from Bego Semados (BEGO Implant Systems GmbH, Germany), 5% (4) implants from Camlog (Camlog GmbH, Wimsheim, Germany) and 4% (3) implants from Pitt Easy (Pitt-Easy^®^, Sybron Implant Solutions, Orange, CA, USA).

### Follow-up examination

All patients were assigned to a fixed clinical follow-up protocol (Fig. 2). Follow-up examinations were performed at seven time points as part of the regular clinical and radiological follow-up examinations following augmentation and dental implant therapies. Prior to the surgical procedure, a detailed preoperative clinical examination was performed (T0) and the indication for the HVSO procedure was individually established. This appointment included a radiographic examination to evaluate primary vertical bone height. After the bone augmentation procedure with HVSO, the gain in vertical bone height was assessed in a postoperative radiographic examination directly post-OP (T1). After three months, another clinical and radiographic examination (T2) was performed to enable implant planning. T3 describes the control directly after implant placement. Implant exposure was performed three months after implantation including a radiographic examination (T4). During the annual implant controls the radiographic follow-up examinations were continued at annual intervals (T5, T6). All patients were re-examined at least up to 1 year after implantation. At the time points after surgery (i.e., after HVSO (T1) and implantation (T3)), complications such as pain, wound healing disorders or paresthesia were documented.

### Assessment of vertical bone gain

**Fig. 3:**
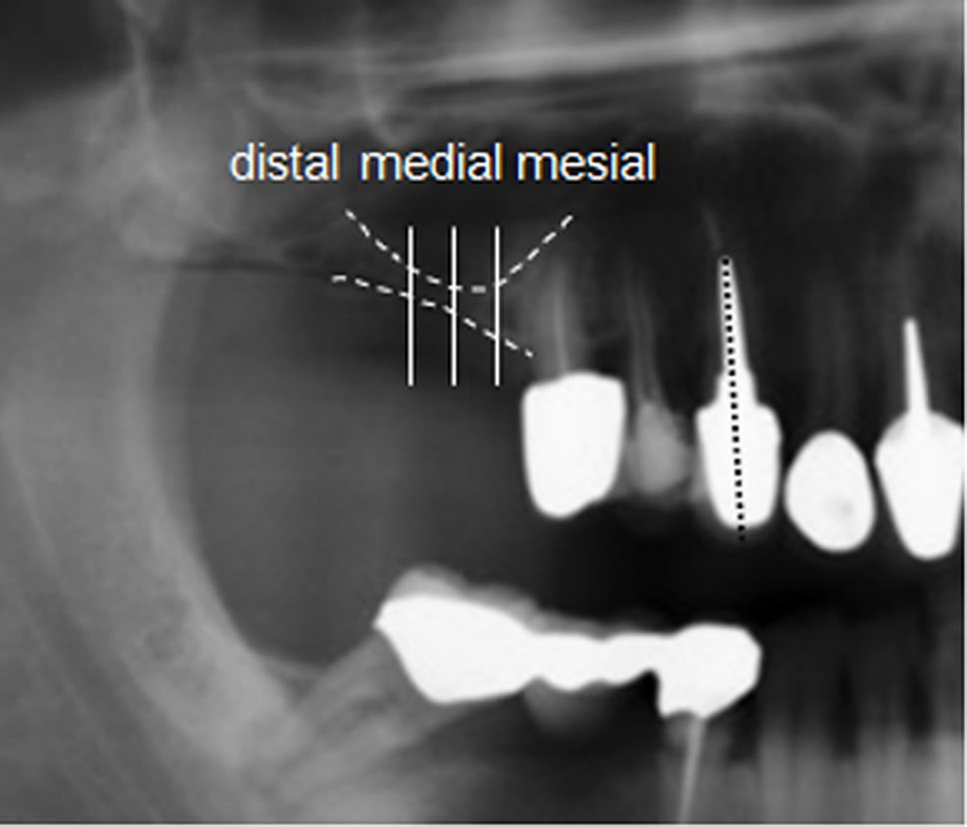
Radiological evaluation technique of the vertical bone height. After marking the bone height in the augmentation area, three reference lines were defined. To avoid image distortion effects in the relevant region, a reference structure was set up. (Here: tooth 13)

Radiographic examinations were used for measuring the vertical bone height at the respective follow-up time points. Radiological follow-up was conducted by means of panoramic tomography (OPTG) using the Orthophos X-ray (Sirona, Bensheim, Germany). A digital analysis of the data was performed with the proprietary software (Sidexis, Sirona, Bensheim, Germany). To define the baseline height, three vertical reference lines were determined in the augmentation area (Fig. 3).

To enhance reproducibility, both vertical lines and the central line of each OPTG were marked at identical locations on structures of consistent size. For consistency purposes, the magnification of all analyzed images remained constant, and all measurements were performed by the same investigator (H.D.), a dentist with extensive experience in radiographic analysis, to ensure reproducibility and accuracy throughout the assessment process.

This approach allowed for the arrangement of images based on these reference lines, facilitating the precise overlay of consecutive images. Measurements of bone heights were then performed at the three marked reference lines. Specifically, in the maxilla, the distance from the most crestal point of the compacta to the floor of the maxillary sinus, and in the mandible to the compacta of the upper edge of the mandibular canal was measured. To ensure consistency, all measurements were conducted by the same investigator (H.B.) who is experienced with radiological dental assessment.

### Statistical analysis

Statistical analysis was performed using IBM SPSS Statistics, Version 21 software (IBM Corp., Armonk, NY, USA). The analysis of changes within the experimental groups over time was calculated using the two-sided dependent T-test, and changes between experimental groups at the time of examination are calculated using the two-sided independent T-test. The correction of Holms-Bonferroni was used to exclude measurement errors in repeated measurements. The long-term survival rate of the implants was analyzed using the Kaplan-Meier method. Statistical significance was defined at a probability level of ≤ 0.05.

## Results

### Study sample

This retrospective study included *n* = 29 patients (14 females, 15 males) with a mean age of 55.4 ± 10.0 years [range: 32–72], which were treated with 34 modified split osteotomies (HVSO) in the maxilla and mandible to allow for dental implantation therapy in the atrophic jaw. (Table 1)

**Table 1:**
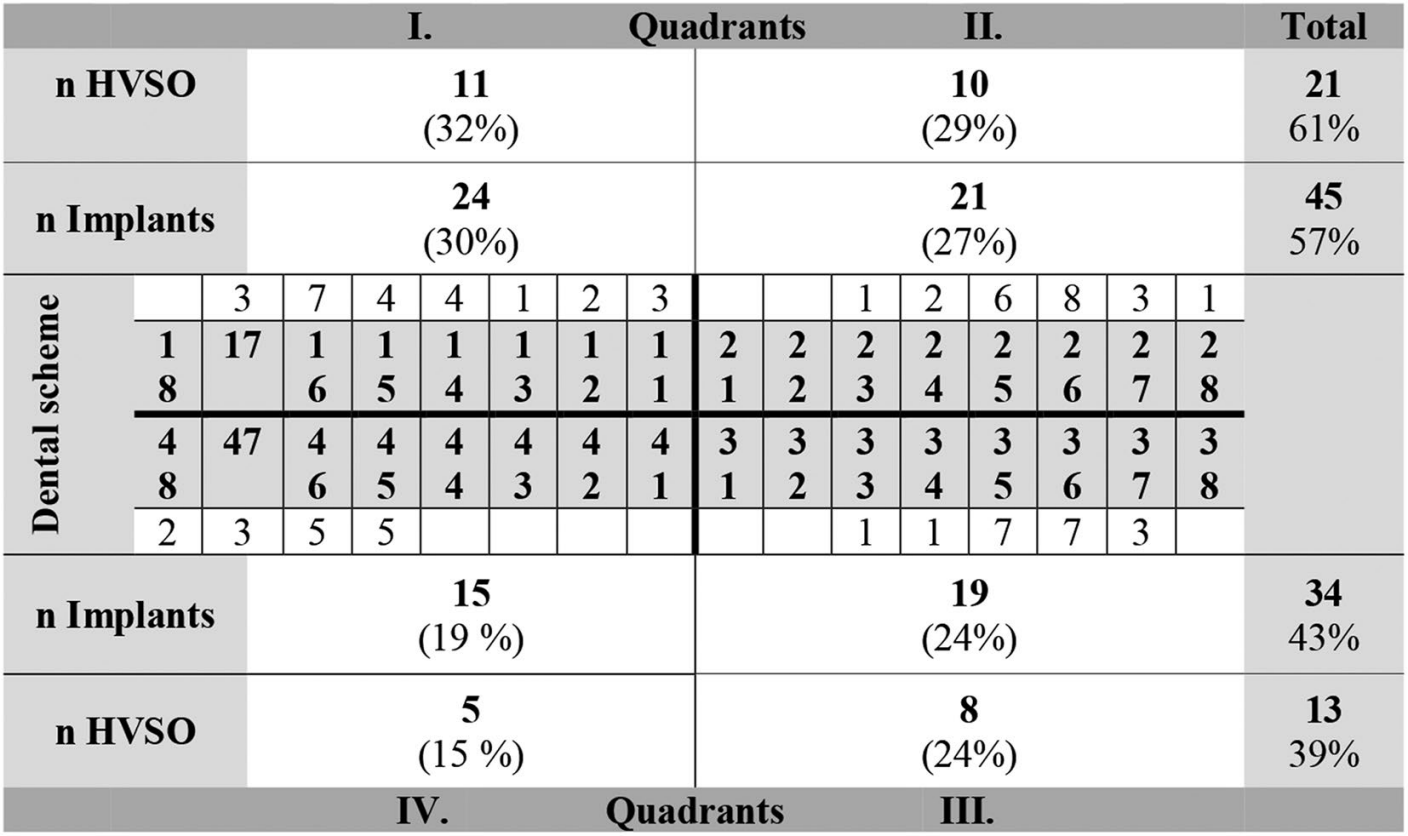
Location and distribution of all HVSO procedures and implant therapies in the study

A total of 79 implants (45 in the maxilla, 34 in the mandible) were placed and clinical evaluation of the implants was performed over a mean follow-up period of 2.3 years. HVSO was performed in a total of 34 quadrants: only one quadrant in 25 patients (eight in quadrant I, seven in quadrant II, five in quadrant III, four in quadrant IV, and one between quadrant III and IV), two quadrants in 3 patients (two in quadrants I and II, and one in quadrants III and IV), while one patient was treated in three quadrants (quadrants I, II, and III). A total of 31 HVSO operations were performed in the posterior region and only three in the anterior jaw region. In four patients the subsequent implant therapy after bone augmentation procedure was performed by the referring dentist. In these four cases bone gain after augmentation was measured. The success of the implant therapy as well as the long-term course were not available since these patients did not show up again for further follow-up examinations.

### Complications and implant loss

Complications were reported in 20.7% of patients (*n* = 6/29 patients), with an overall implant loss rate of 8.9% (*n* = 7/79 implants). Two implants were lost in one patient due to an infection of the augmentation material, and one implant loss was associated with transient paresthesia. Four patients experienced isolated implant loss without any associated complications. Specifically, five implants were lost before exposure (6%), one implant was lost due to loosening after one year (2.5%), and another was lost after more than three years (2.5%).

### Vertical bone gain

The radiographically assessed mean preoperative bone height at time point T0 was 7.5 ± 5.1 mm [range: 1.9–21.0], with significant variations between the preoperative bone height in the maxilla and the mandible with 5.1 ± 3.7 mm and 11.6 ± 4.2 mm, respectively. Despite the high mean values and standard deviations, three-quarters of the patients showed a preoperative vertical bone height of less than 6.0 mm in the maxilla and less than 13.0 mm in the mandible (Table 2).

Directly after the HVSO procedure (T1), the vertical bone height was augmented on average from 7.5 ± 5.2 mm to a total of 12.0 ± 4.2 mm in the maxilla and mandible with an average bone gain of + 4.4 mm ± 2.0 mm (+ 59.4%), with *p* < 0.001. Specifically, a statistically significant vertical bone augmentations of + 5.2 ± 1.9 mm (+ 101.0%) and + 3.2 ± 1.3 mm (+ 27.5%) were achieved in the maxilla and mandible with *p* < 0.001, respectively. These differences in bone level were also significant after Bonferroni-Holm correction.

**Fig. 4:**
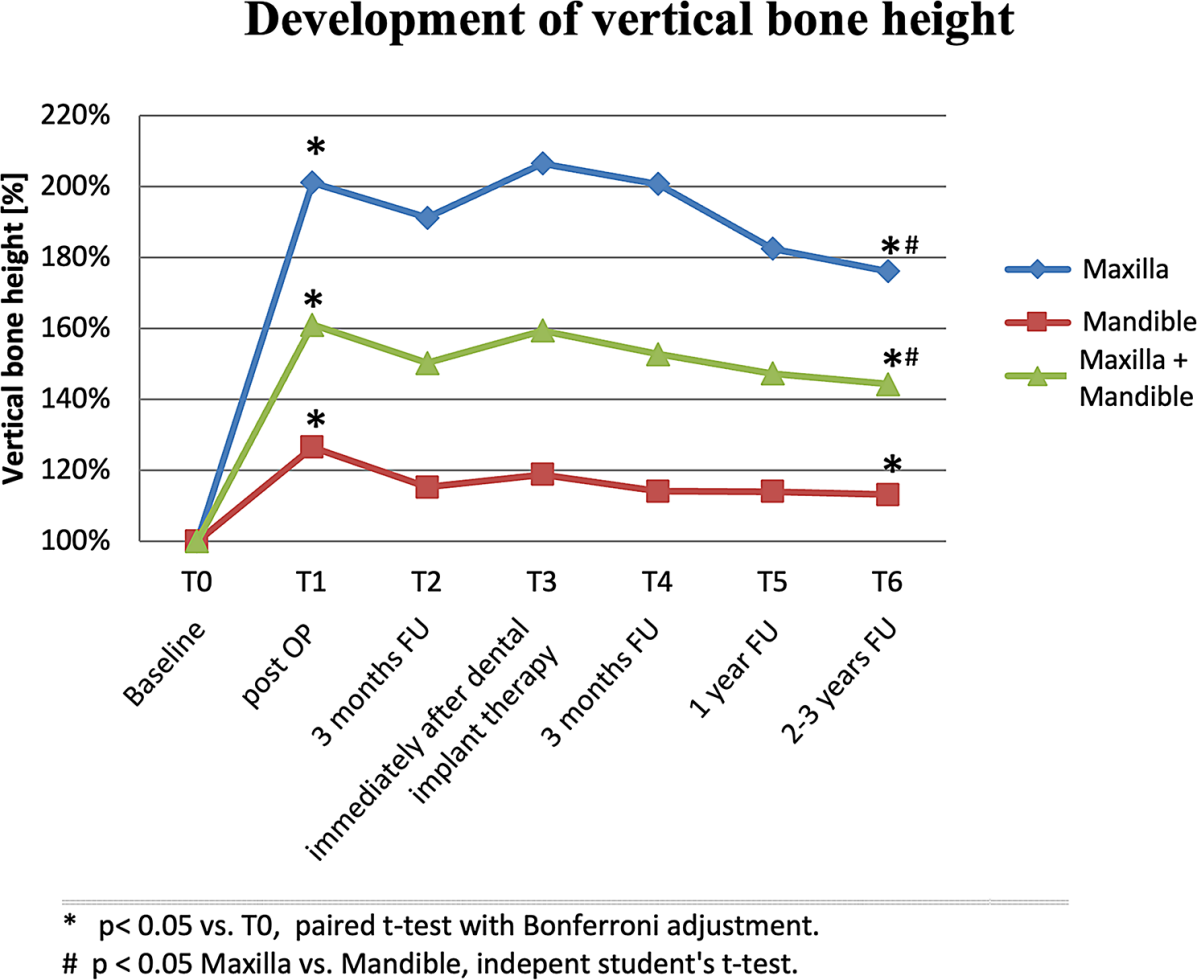
Relative development of vertical bone height in the maxilla and mandible over the follow-up period of 2.3 years

**Fig. 5:**
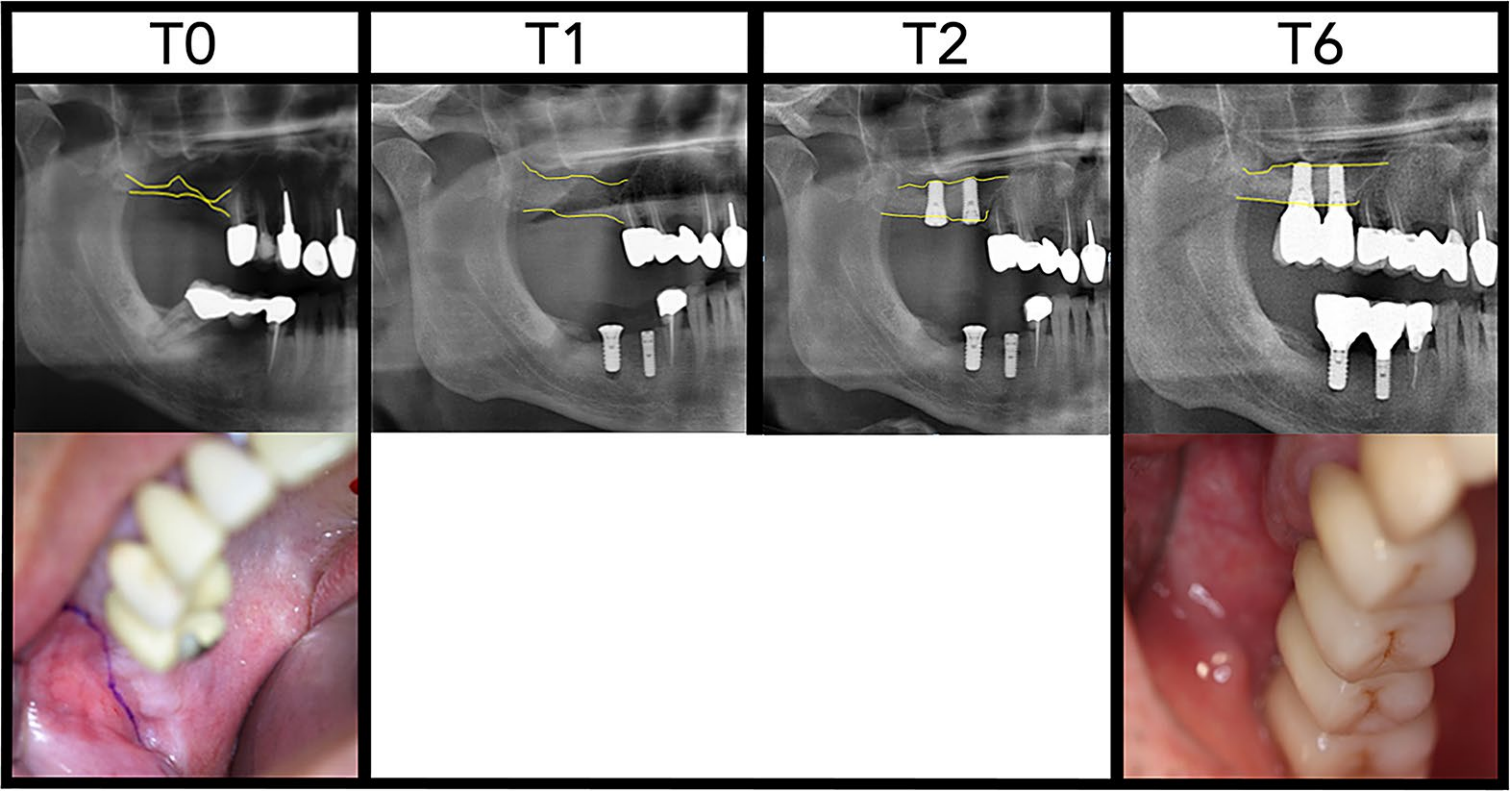
Clinical case of a 58-year-old patient with severe maxillary bone atrophy in the first quadrant. Initial clinical and radiological assessment showed a residual bone height (yellow lines) of about 1 mm with a vertical deficit and bite height loss of 4–5 mm (T0). Immediately after HVSO, bone height was augmented to approximately 9 mm (T1). After a three-month healing period, two Straumann implants (4.1 × 10 mm) were placed in regions 16 and 17 (T2). At 3.5 years post-implantation, bone stability around 8 mm is observed, with crown restorations maintaining a near-physiological crown-to-root length ratio of 1:1 (T6)

At the last follow-up time point (T6), with an average of 2.3 years after implantation, the mean value of bone height in both jaws was 10.9 ± 3.6 mm in total. Thus, a statistically significant increase in vertical bone height of + 3.1 ± 2.3 mm (+ 41.4%) was achieved with *p* < 0.001. The final bone heights were 9.05 ± 2.0 mm in the maxilla and 13.1 ± 4.0 mm in the mandible with an increase of + 3.7 ± 2.6 mm (+ 72.6%) and + 2.2 ± 1.6 (+ 18.6%), and *p* < 0.001 and *p* = 0.001, respectively. (Table 2; Figs. 4 and 5)

**Table 2:**
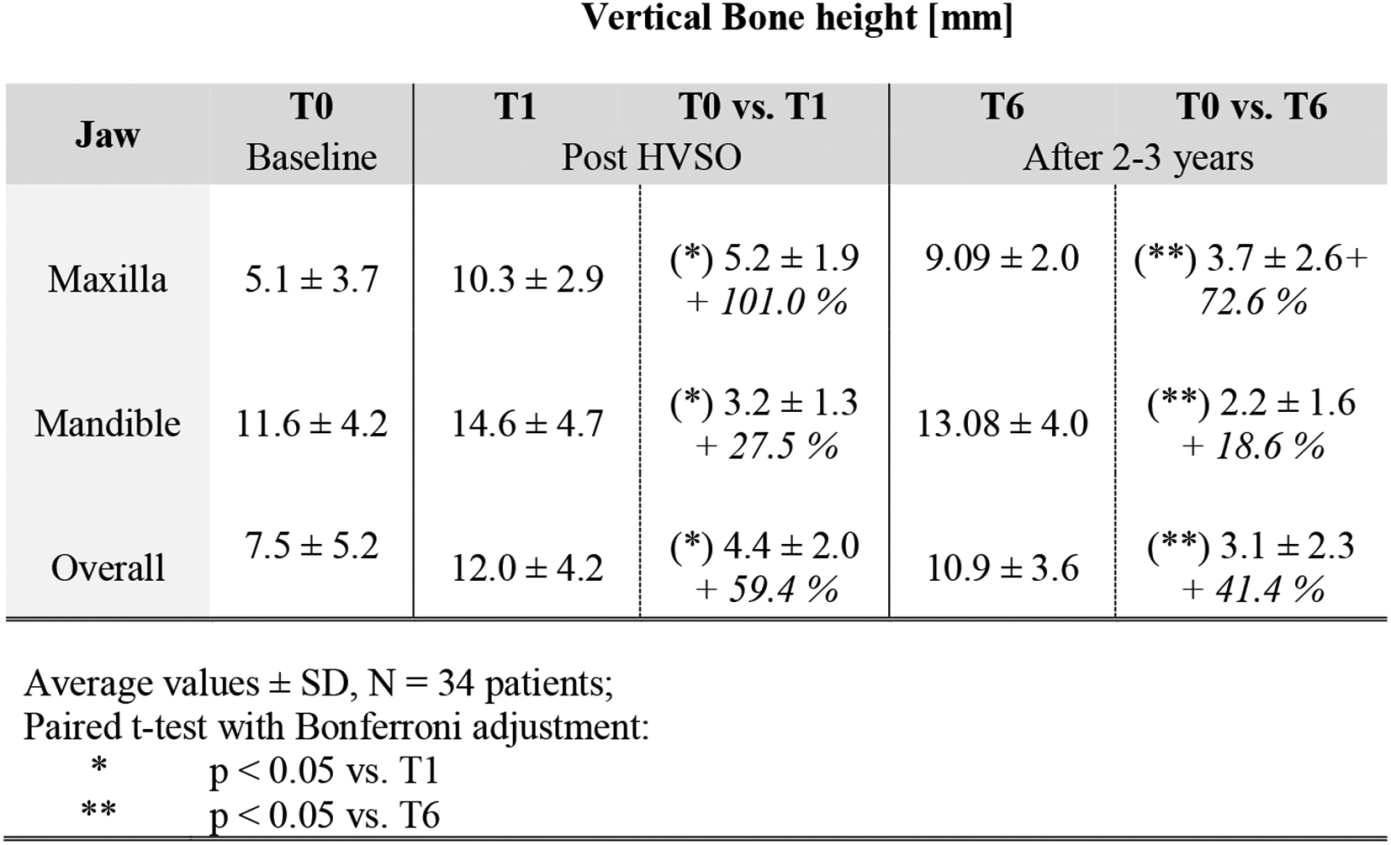
Average bone height measurements at times T0, T1, and T6 with their absolute and relative gain at T1 and T6

### Implantation survival

**Fig. 6:**
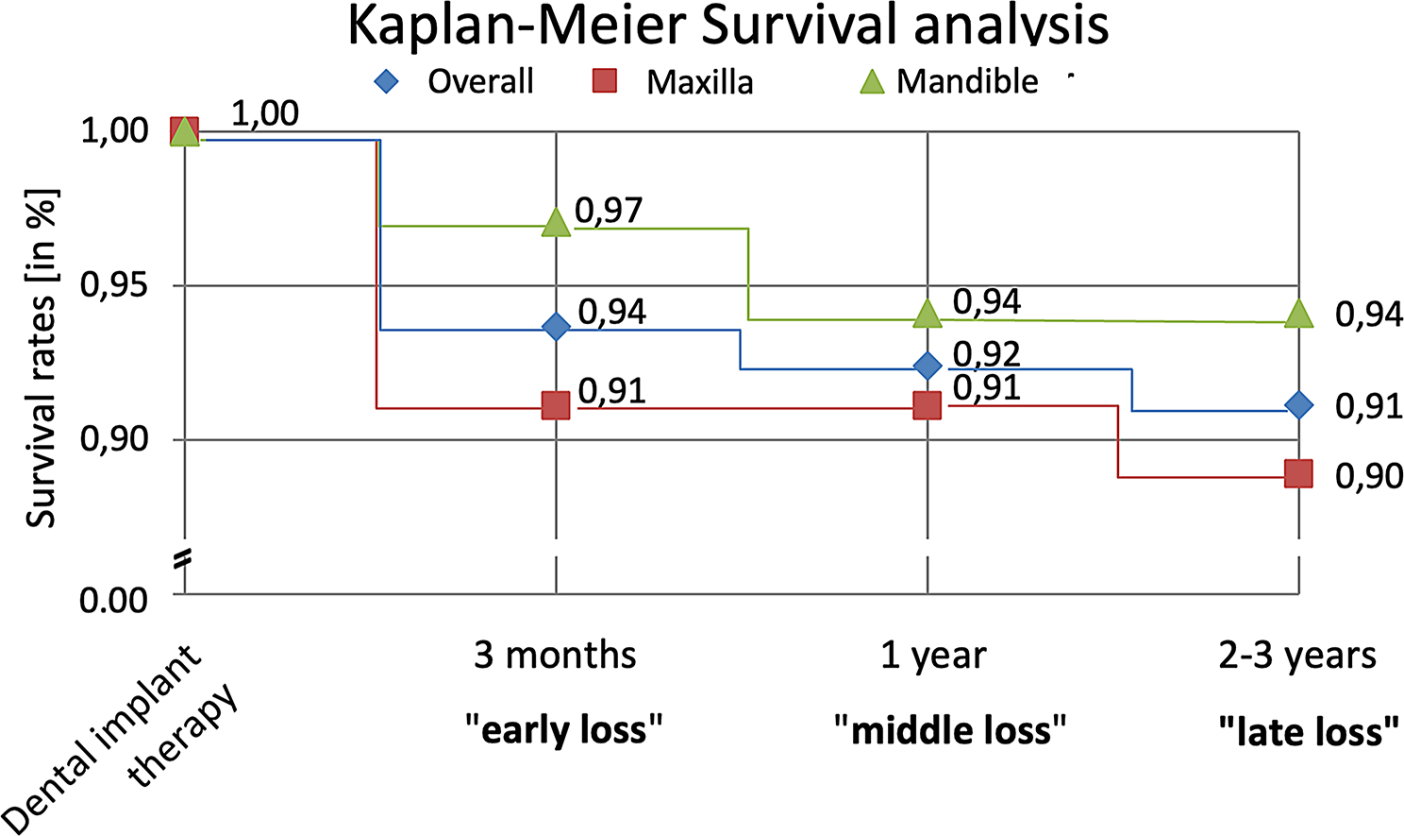
Kaplan-Meier survival analysis of the dental implants in this study

Over the mean observation period of 2.3 years after implantation, 91.1% (72/79) of the placed implants remained stable clinically and radiologically. (Fig. 6).

Stratified by jaw, the survival rates of implants were 94.1% in the maxilla and 88.9% in the mandible. In the maxilla, five of the 45 implants (11.1%) were lost, of which two implants were lost due to infection of the graft in one patient with poor oral hygiene. In the mandible, two of the 34 implants (5.8%) were lost in one patient with history of heavy tobacco use and poor oral hygiene. Five of the seven implant failures occurred as early failures before prosthetic restoration. Four of the five lost implants received successful implant therapy after bone regeneration. In one case a sufficient prosthetic restoration was still possible, therefore another implant therapy was not necessary. The survival rate for the 54 Straumann implants was 92%, with five implants lost. Conversely, two of the 13 Bego implants (15%) were lost in a single patient with poor oral hygiene and a history of severe pipe smoking. There were no failures reported with Camlog and Pitt Easy implants. All complications observed in our study are summarized in Table 3.

**Table 3:**
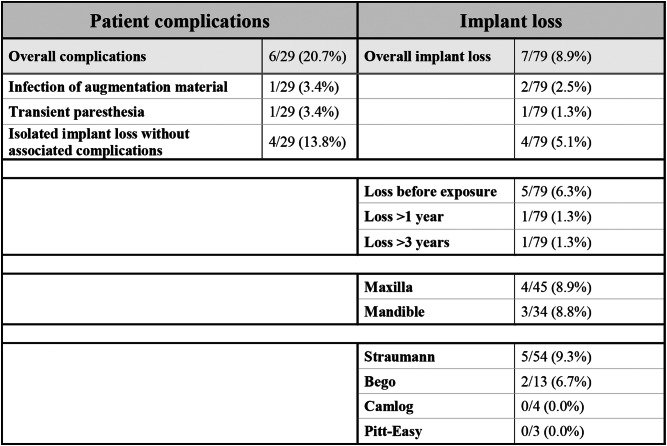
Summary of complications observed in our study

## Discussion

This study reports on the Horizontal Vascular-Stalked Split Osteotomy (HVSO), an innovative approach to jawbone augmentation, particularly for patients with significant alveolar ridge deficiencies. The aim was to evaluate the efficacy of HVSO in enhancing vertical bone height and supporting dental implant therapy in patients with severe jaw atrophy. The results demonstrate promising outcomes, with notable increases in vertical bone height and comparatively low complication rates.

Interestingly, this investigation revealed a statistically significant higher increase in vertical bone height in the maxilla compared to the mandible. More specifically, the maxilla showed a 101.0% increase at T1 and 72.6% at T6, whereas the mandible exhibited a 27.5% increase at T1 and 18.6% at T6. One may hypothesize a series of reasons behind this jaw-dependent discrepancy in vertical bone height increases: Firstly, the initial vertical bone heights in the maxilla were significantly lower than those in the mandible. Preoperative bone heights averaged 5.1 mm in the maxilla compared to 11.6 mm in the mandible. This baseline disparity likely contributed to the more substantial relative increases observed in the maxilla. In other words, the greater deficiency in the maxilla provided more room for improvement, thus amplifying the percentage increase post-augmentation. Secondly, the anatomical and biological differences between the maxilla and mandible play a crucial role. The maxilla, being less dense and more vascularized, might respond more favorably to augmentation procedures. In addition, the upwardly open maxillary sinus provides a relatively spacious cavity that facilitates the accommodation of graft materials and subsequent bone growth. In contrast, the denser cortical bone of the mandible and the presence of the mandibular canal limit the space available for vertical augmentation. Thirdly, the biomechanical forces acting on the maxilla and mandible differ significantly. The maxilla experiences less stress and mechanical loading than the mandible due to its positioning and the distribution of masticatory forces which can impact the stability and integration of grafted bone material. The stress and strain on the augmented bone in the mandible may inhibit the overall vertical bone gain, as the bone remodeling process is constantly influenced by these mechanical forces impressing the elevated bone segment. Lastly, the different biological environments in the two jaws could influence the healing and regeneration processes. The increased vascularity supports better delivery of nutrients and osteogenic cells, thereby promoting more effective bone regeneration and augmentation. In contrast, the relatively avascular nature of the mandible combined with a considerable application of force, may hinder the healing process, resulting in less effective augmentation [26–28]. 

Irrespective of the jaw investigated, radiographical measurement yielded significant overall increases in vertical bone height by + 4.4 mm (+ 59.4%) immediately after the surgical procedure (T1) and by + 3.1 mm (+ 41.4%) after a mean observation period of 2.3 years. Comparing these findings to the existing literature, we conclude that both the immediate efficacy and the longevity of bone height augmentation with HVSO appear to be promising. In fact, studies on traditional jawbone augmentation techniques report varying degrees of bone resorption over time [29–32]. Traditional bone grafting techniques are often linked with higher bone resorption rates, whereas this study showed a relatively stable bone height with only a minor reduction over time. The herein noted sustained bone height can be attributed to several factors: HVSO is a technique characterized by a less traumatic approach compared to traditional jawbone augmentation procedures, emphasizing the preservation of the lingual and/or palatinal vascular supply, which is crucial for the effective and long-lasting integration of the bone augmentation material. The significance of maintaining vascular integrity, as emphasized by the HVSO technique, finds support in the broader literature, which consistently reports better outcomes in procedures that minimize vascular disruption [33–35]. This principle is crucial for ensuring effective integration of augmentation materials and long-term stability of the augmented bone, a factor often overlooked in conventional jawbone augmentation techniques. In short, the preservation of vascularity in the stalked bone fragments likely enhances the integration and stability of the graft materials, reducing the risk of resorption and warranting long-term sustained results. In addition, the minimally invasive nature of the HVSO technique reduces trauma and promotes better healing, contributing to the stability of the augmented bone. Regarding the dimensions of the mobilized bone segment, spanning at least two missing teeth has proven clinically advantageous to ensure adequate width and stability. Single tooth gaps are unsuitable for this technique due to the increased risk of damaging adjacent tooth roots with the piezo saw. The minimum recommended width of the mobilized bone segment is approximately 1.5 cm.

These procedural advantages of HVSO are also reflected in a relatively low complication rate of 20.7%, which falls within the lower range when compared to other vertical ridge augmentation techniques previously reported. In a recent systematic review, Sáez-Alcaide et al. recorded a complication rate of 38.01% for bone block procedures, which is significantly higher than our findings. Specifically, the most frequent complications in their review were paresthesia (21.8%), followed by graft exposure (10.17%) and graft loss (2.4%). Herein, we only documented two cases of transient paresthesia and an overall implant loss rate of 8.9% which was easily resolved by an implant procedure at the second attempt. Osteogenesis distraction was also found to be associated with a significantly higher risk of postoperative morbidity, with– according to the pooled analysis by Sáez-Alcaide et al.– more than one in two patients experiencing any adverse events. Frequent issues included paresthesia (11.2%), misdirections (8.16%), acute mucosa inflammation (6.12%), and segment tilting (6.1%) [36, 37] Investigating clinical outcomes of vertical distraction osteogenesis for dental implantation, Zhao et al. even documented a complication as high as 82%. Importantly, in light of these high complication risks, the authors called for operative caution. For guided bone regeneration, Sáez-Alcaide et al. noted a complication rate of 16.8%, with membrane exposure being the most common issue (10.9%). Similar results were also found in the systematic review by Urban et al., which analyzed the effect of various techniques used for vertical ridge augmentation on clinical vertical bone gain: Distraction osteogenesis achieved the highest mean gain of 8.04 mm, but had a high complication rate of 47.3%, while GBR had a mean gain of 4.18 mm and a lower complication rate of 12.1%. Bone blocks showed a mean gain of 3.46 mm with a complication rate of 23.9% [36].

In light of these data, the technique presented herein showed favorable complication rates, while the nature of complications also differed. HVSO complications were more related to transient paresthesia and isolated implant losses without associated membrane issues. The preservation of the periosteal stalk in HVSO might reduce the risk of membrane-related complications, though this influence needs to be further corroborated in future studies. The high complication rates associated with bone blocks and distraction osteogenesis are testament to the invasive nature of these procedures and the technical difficulties inherent to their execution. In contrast, the lower complication rates observed with HVSO highlight its potential as a safer alternative for bone augmentation in atrophic jaws. In this context, it is also worth mentioning that the variation in complication rates across trials reveals the need for standardized reporting criteria to facilitate more accurate comparisons and improve clinical decision-making.

### Limitations

This study’s findings ought to be interpreted in light of its inherent limitations. The small sample size of *n* = 29 patients and the lack of a control group might limit the generalizability of the findings presented herein. Additionally, the retrospective nature of the study may introduce biases related to data collection and analysis. The study also did not consider potential variations in surgical technique or post-operative care that could influence outcomes. Future research is required to further validate the HVSO technique through long-term prospective studies and comparative analyses with other augmentation methods, thereby providing a more in-depth understanding of HVSO’s potential benefits over traditional techniques, especially in terms of minimizing postoperative morbidity and enhancing patient outcomes.

## Conclusion

This study demonstrates that the Horizontal Vascular-Stalked Split Osteotomy (HVSO) is an effective and reliable surgical technique for achieving vertical bone augmentation in severely atrophic jaws. By enabling stable long-term outcomes and satisfying implant survival rates, this technique facilitates successful dental implant therapy in cases that would otherwise be unsuitable for conventional approaches.

## Data Availability

The research data is available from the corresponding author upon reasonable request.

## Abbreviations

HVSO: Horizontal Vascular–Stalked Split Osteotomy
IRB: Institutional Review Board
OPTG: Orthopantomogram
OP: Operation
SD: Standard Deviation
SPSS: Statistical Package for the Social Sciences
mm: Millimeters
